# Dietary Factors and Supplements Influencing Prostate-Specific Antigen (PSA) Concentrations in Men with Prostate Cancer and Increased Cancer Risk: An Evidence Analysis Review Based on Randomized Controlled Trials

**DOI:** 10.3390/nu12102985

**Published:** 2020-09-29

**Authors:** Maria G. Grammatikopoulou, Konstantinos Gkiouras, Stefanos Τ. Papageorgiou, Ioannis Myrogiannis, Ioannis Mykoniatis, Theodora Papamitsou, Dimitrios P. Bogdanos, Dimitrios G. Goulis

**Affiliations:** 1 Department of Rheumatology and Clinical Immunology, Faculty of Medicine, School of Health Sciences, University of Thessaly, Biopolis, GR-41334 Larissa, Greece; mariagram@auth.gr (M.G.G.); bogdanos@med.uth.gr (D.P.B.); 2 Medical School, Faculty of Health Sciences, Aristotle University of Thessaloniki, University Campus, GR-54124 Thessaloniki, Greece; papagesn@auth.gr (S.Τ.P.); ioannismyrogiannis@outlook.com (I.M.); 3 Institute for the Study of Urological Diseases (ISUD), 33 Nikis Avenue, GR-54622 Thessaloniki, Greece; g_mikoniatis@hotmail.com; 4 1st Department of Urology and Center for Sexual and Reproductive Health, G. Gennimatas—Aghios Demetrius General Hospital, 41 Ethnikis Amynis Street, Aristotle University of Thessaloniki, GR-54635 Thessaloniki, Greece; 5 Laboratory of Histology and Embryology, Medical School, Faculty of Health Sciences, Aristotle University of Thessaloniki, GR-54124 Thessaloniki, Greece; thpapami@auth.gr; 6 Division of Transplantation, Immunology and Mucosal Biology, MRC Centre for Transplantation, King’s College London Medical School, London SE5 9RS, UK; 7 Unit of Reproductive Endocrinology, 1st Department of Obstetrics and Gynecology, Medical School, Faculty of Health Sciences, Aristotle University of Thessaloniki, GR-56429 Thessaloniki, Greece

**Keywords:** malignancy, obesity, benign prostate hyperplasia, prostatic intraepithelial neoplasia, dietary supplements, polyphenols, genistein, resveratrol, sulforaphane, *Serenoa repens*

## Abstract

The quest for dietary patterns and supplements efficient in down-regulating prostate-specific antigen (PSA) concentrations among men with prostate cancer (PCa) or increased PCa risk has been long. Several antioxidants, including lycopene, selenium, curcumin, coenzyme Q10, phytoestrogens (including isoflavones and flavonoids), green tea catechins, cernitin, vitamins (C, E, D) and multivitamins, medicinal mushrooms (*Ganoderma lucidum*), fruit extracts (saw palmetto, cranberries, pomegranate), walnuts and fatty acids, as well as combined supplementations of all, have been examined in randomized controlled trials (RCTs) in humans, on the primary, secondary, and tertiary PCa prevention level. Despite the plethora of trials and the variety of examined interventions, the evidence supporting the efficacy of most dietary factors appears inadequate to recommend their use.

## 1. Introduction

Prostate-specific antigen (PSA) consists of a glycoprotein excreted by both healthy and cancerous cells, with the latter indicating an increased risk for prostate cancer (PCa). Identifying men most likely to harbor PCa is important for early diagnosis and better prognosis [1]. PSA screening has significantly increased the number of men treated for PCa, and it has been estimated that in ten years, many of them would remain asymptomatic, without experiencing PCa-specific mortality [2]. According to a recent meta-analysis, PSA screening was shown to produce a small reduction in 10-year disease-specific mortality, without any benefits towards overall mortality [3]. As a result, most clinical practice guidelines do not advocate for a prostate biopsy based on elevated PSA concentrations alone [1,2,4], whereas based on the available evidence, the use of opportunistic routine PSA screening is also not recommended for all [1,2,5]. Nevertheless, elevated PSA concentrations usually require a second sample for verification purposes, whereas persistent elevated PSA concentrations, are usually followed by prostate biopsy and diagnostic imaging [2,6].

Since the antigen is not PCa-specific and can be affected by various factors, caution is required before relying on its diagnostic accuracy. It has been suggested than one in seven men undergoing PSA screening will demonstrate increased concentrations [2,7,8]. As a result, a controversy was sparked among experts on the usefulness of routine PSA screening. Some suggest that the use of PSA might reduce the relative risk for PCa by 9% [8], whereas others highlight its low impact on mortality, as well as the drawbacks and harms associated with PCa overdiagnosis and overtreatment [2,3,9,10,11], including urinary incontinence, erectile dysfunction, and a possibly reduced quality of life [12,13]. However, for some researchers, even the increased PCa prevalence observed during the latest quarter of the 21st century is the epiphenomenon of extended PSA screening [14,15].

On the other hand, it has been suggested that approximately 35% of the men treated for PCa will experience at least one biochemically defined recurrence, as indicated by a rise in PSA concentrations, within ten years of local therapy [16]. Although several factors have been shown to contribute to this phenomenon [17], the effect of diet on PSA concentrations is not crystal clear. In parallel, apart from PCa, benign prostate hyperplasia (BPH), although a common finding among men with increasing age [18], is also associated with increased PSA concentrations and according to meta-analyses, it often consists of a precursor to PCa development, as it is associated with an increased PCa risk [19].

Research has shown that both BPH and PCa patients often resort to dietary manipulations, complementary and alternative medicine (CAM), and/or nutritional supplements to lower PCa risk, through the reduction of PSA concentrations [20,21,22,23]. However, the evidence regarding the efficacy of dietary manipulations and nutritional supplements on reducing PSA concentrations among men with PCa and/or BPH is scattered [24]. To fill this gap, the present review aimed to gather all evidence on the effects of diet and dietary supplements on PSA concentrations among men with PCa and BPH, to aid the formulation of recommendations regarding their use. Evidence from the higher steps of the evidence-based pyramid, being randomized controlled trials (RCTs) and meta-analyses were deployed to evaluate the effectiveness of nutritional factors in down-regulating PSA concentrations.

## 2. Obesity and Serum PSA Concentrations

Overall, cross-sectional research is unanimous on the fact that increased body mass index (BMI) is associated with lower PSA concentrations [25,26,27]. According to a recent systematic review and meta-analysis using 35 primary studies, each increase in BMI by 5 kg/m^2^ is associated with a reduction in PSA concentrations equal to 5.88% (95% CI −6.87 until −4.87) [28]. On the other hand, BMI did not appear to have any association with PCa or advanced PCa risk [28]. A rise in PSA concentrations has been reported to occur post-weight loss, as the epiphenomenon of hemoconcentration, while on the other hand, a reduction in PSA is experienced with increasing body weight, due to hemodilution [29]. As this effect might influence PCa diagnosis, it has been suggested that PSA concentrations of overweight men should be corrected accordingly and multiplied by 3.5%, whereas when obese men are concerned, multiplication by 13% is required to attain more accurate results [27,30].

Three RCTs assessed the efficacy of pre-surgical weight-loss on PSA concentrations among men with PCa however, the first failed to induce any significant changes in either the circulating PSA or the BMI of participants [31] over a 50-days lifestyle intervention. The second and third RCTs consisted of the feasibility [32] and pilot study, respectively [33], without any PSA results being reported in either. Nevertheless, in the first RCT [31], although a tendency for increased PSA concentrations was noted among intervention participants, in parallel, Ki67 proliferation rates were also greater post-intervention compared with the controls, indicating that more studies are required before recommending rapid weight loss among obese patients with PCa [31]. Nevertheless, one RCT comparing a 6-week caloric restriction vs. standard diet among overweight men with newly diagnosed PCa did not report any change in PSA concentrations [34].

As for BPH, according to a preponderance of published evidence, it has been suggested that obesity and metabolic syndrome markedly increase prostatic tissue inflammation, prostate volume, and prevalence of BPH [35,36,37]. In parallel, a positive dose–response relationship has been identified between BMI and BPH, as reported by a meta-analysis of case-control studies [38]. Therefore, by inference, when BPH and increased body weight are apparent, PSA concentrations are reduced due to the aforementioned hemodilution phenomenon, and for this, RCTs should focus on recruiting patients with similar BMIs to correct for this phenomenon. 

## 3. Antioxidants and PSA Kinetics

Plenty of RCTs have assessed the effects of selected antioxidant supplementation in circulating PSA concentrations. Given that cancers and PCa, in particular, are promoted by reactive oxygen species (ROS), men with PCa have been shown to exhibit upregulated oxidative stress and impaired antioxidant defense systems [39]. As a result, antioxidant oral nutrient supplement (ONS) has been suggested as an adjuvant therapy to correct these observations and possibly act as an anti-carcinogen. Vitamins C, E, and D, lycopene, selenium, curcumin, flavonoids, catechins, fruit and fruit extracts have all been studied and postulated to affect PSA concentrations; however, in the vast majority, evidence from existing RCTs appears inconsistent [40] and does not seem to support the use of antioxidants.

### 3.1. Lycopene

Lycopene is a carotenoid compound, found in high doses in the tomato fruit and tomato-based products [41]. Meta-analyses have suggested that frequent lycopene intake can reduce cancer-related symptoms, whereas, on the other hand, before the diagnosis, high circulating serum lycopene concentrations were associated with a reduced PCa risk [42,43]. With this in mind, it was suggested that the intake of nutrients protecting against cancer development might also entail anti-carcinogenic effects post-diagnosis, by halting disease progression [44,45]. This stance was supported by longitudinal studies indicating that specific PCa harboring *TMPRSS2* appears mediated by increased tomato intake [46]. As a result, lycopene and tomato products were extensively examined in PCa research (Table 1).

Among patients with PCa scheduled for orchidectomy, comparison of PSA concentrations among those receiving lycopene versus those undergoing orchidectomy only failed to reveal any differences in the PSA concentrations [47]. Nevertheless, a greater PSA response has been reported among men receiving lycopene [47,48], although this effect might involve a transient response, not specific to lycopene [48]. When newly diagnosed men scheduled for prostatectomy were recruited [49], supplementation with a tomato oleoresin extract containing 30 mg of lycopene until operation significantly reduced tumor size and PSA concentrations compared with no intervention. In a similar double-blind trial, however, Kumar et al. [50] failed to induce a notable reduction in the PSA of men receiving escalating lycopene doses, compared with the controls. According to an old systematic review [42] of randomized and non-randomized clinical trials, an inverse association between lycopene intake and PSA concentrations is apparent in most trials using men with PCa as their sample population, however, the great heterogeneity observed in supplementation regime and design of the included trials did not allow for a synthesis of the evidence. More recently, Ilic [51] attempted a similar systematic review using RCTs as the only primary studies and reported a reduction in PSA concentrations among men with PCa receiving lycopene. 

In a Cochrane version of a similar research question however [53], no difference was determined in the circulating PSA concentrations of men with PCa receiving lycopene, as compared with those randomized to a placebo supplement.

In the primary prevention level, Schwarz et al. [52] failed to induce any differences in PSA concentrations of men with histologically proven BPH, free of PCa. Additionally, a Cochrane systematic review and meta-analysis [53] also failed to report any statistical difference in PSA concentrations between men receiving lycopene compared with the controls. This lack of efficacy observed among studies on humans is also corroborated by systematic reviews of in vitro and animal studies [54], revealing that for the time, lycopene appears to be more of hype rather than hope for PCa.

Overall, lycopene supplementation RCTs are highly heterogeneous in terms of design, included subjects, and administered dosage [53], with most of the reported significant effects of lycopene on PSA concentrations stemming from observational studies [55]. According to a recent systematic review of in vivo and in vitro studies, lycopene supplementation appears to have null, or little effect on androgen-related outcomes [54] due to the inconsistent findings of the primary studies [56]. Moreover, a meta-analysis of case-control studies failed to suggest the existence of a relationship between high serum lycopene concentration and PCa risk [56].

### 3.2. Selenium

Selenium, an essential trace element and a constituent of more than 25 selenoproteins neutralizing free radicals, has been examined as an anti-cancerogenic agent since 1949 [40,57]. Meta-analyses of case-control studies have revealed an inverse PCa risk among men with high serum selenium concentrations, suggesting a possible protective role [58,59].

In PCa-related RCTs, selenized yeast appears to be the preferred form of supplementation. Algotar et al. [60] examined the effect of selenium supplementation among men at high risk for PCa (Table 2). His findings revealed that selenium supplementation did not affect the PSA concentrations or the incidence of PCa through a 5-year spectrum. This lack of efficacy on the primary prevention level was also verified in more recent studies, examining longitudinal selenium supplementation among apparently healthy men [61,62] and meta-analyses [63]. Considering the dose–response association of all antioxidant supplements, according to another meta-analysis [64], selenium ONS might protect against PCa in the low baseline serum selenium concentration population only.

Moreover, when men with localized PCa were used in a similar trial, in the highest quartile of baseline selenium, men supplemented with a greater dose experienced the higher PSA velocity as compared with the placebo group, indicating that high-dose selenium ONS appears to be a risk factor for increased PSA velocity among men with high baseline plasma selenium concentrations [65] (Table 2). This finding reveals that selenium ONS is not risk-free and might increase the risk for PCa if taken over-the-counter, uncontrollably. As far as PCa mortality is concerned, according to prospective studies, supplementation with 140 μg/day or more post-diagnosis of non-metastatic PCa may augment the risk of PCa mortality [66]. These findings, indicating that high-dose selenium supplementation might, in fact, pose a greater PCa risk, are explained by the inverted U-shape effect reported during antioxidant supplementation [67,68]. Nevertheless, the intake of selenium from foods and supplements should not exceed 400 mg daily. In contrast, in the Stratton [65] trial, one of the treatment arms was consuming twice this amount for up to 5 years, indicating that selenium overload might have occurred in some participants, reversing the result from efficacy to non-efficacy, based on the U-shape response. For this, unsupervised supplementation is not warranted [69], whereas in cases of prescribed ONS, the dose should be tailored to the serum concentrations based on frequent checks, to avoid triggering a negative effect.

### 3.3. Curcumin

Curcumin is a naturally-derived pleiotropic compound, targeting diverse signaling and molecular pathways [70]. Curcumin derives from the turmeric plant and has been used as an adjuvant therapy to many diseases requiring a boost of the immune system [71,72]. Moreover, curcumin has been suggested to entail chemopreventive and anticancer properties related to PCa [73,74]. Two placebo-controlled trials (Table 3) have compared curcumin ONS among patients with PCa, both failing to induce any differences in circulating PSA after 3 [75] and 6 [76] months of supplementation, respectively.

### 3.4. Phytoestrogens

Phytoestrogens are bioactive molecules, including various structurally distinct plant compounds included mainly in vegetables [77]. Phytoestrogens have been suggested to affect reproduction on many levels, including the prostate gland [78]. The four main compounds of phytoestrogens are isoflavonoids, flavonoids, stilbenes, and lignans [79].

Flavonoids are phenolic substances occurring in plants that retain radical-scavenging ability even after ingestion [80]. The flavonoid family encompasses more than 8000 distinct compounds [80], with isoflavones, mainly present in soy products, being the most examined in in vivo cancer research. Physiology-wise, flavonoids have been suggested to target oncogenes and tumor suppressor genes, while restoring both miRNA and lncRNA expression, which is altered during disease progress [81]. Although individual RCTs might advocate for the efficacy of flavonoids in reducing PSA concentrations and PCa risk, a 2014 meta-analysis of RCTs indicated that the consumption of soy products or isoflavones did not induce any significant differences in the PSA concentrations between the control and intervention group among men with PCa diagnosis or a clinically identified risk of PCa [82]. 

Genistein is a major isoflavone acting as a chemotherapeutic agent in various cancer cells, modulating cell angiogenesis, apoptosis, and metastasis [83]. Among men with localized PCa scheduled for radical prostatectomy (Table 4), supplementation with 30 mg of synthetic genistein until the operation failed to reduce PSA concentrations of participants, compared with a placebo [84,85]. It appears however, that this lack of genistein efficacy in PCa in vivo studies is even extended in non-RCTs, with genistein ONS presenting null efficacy [86].

Bylund [87] compared rye bran with wheat bread consumption among men with conservatively treated PCa and failed to report significant changes between intervention arms. When men with PCa were randomized to consume whole grain and bran products, substituting 50% of their daily total energy intake (TEI), or refined wheat products with added cellulose (50% of TEI) in a cross-over trial, no differences were recorded in the PSA change from baseline between treatment arms [88].

Using bread again as a medium for supplementation, Dalais et al. [89] examined the effect of high vs. low phytoestrogen content. Participants consumed bread supplemented with 50 g of heat-treated (HT) soy grits, bread with a similar soy grits content plus 20 g of linseed, or wheat bread instead as control, until the date of radical prostatectomy operation. The percentage change in total PSA and free/total PSA ratio differed between the HT soy grits arm and the control group, indicating some efficacy [89]. In a similar phase II trial, Kumar [90] failed to produce PSA changes following aglycone isoflavones ONS (40 mg), compared with placebo, until the date of radical prostatectomy.

When escalating doses of isoflavones were assessed in a phase I placebo-controlled trial, the lack of efficacy was still apparent from biopsy to prostatectomy [91]. This lack of effect was even evident in double-blind RCTs of greater duration [92,93], suggesting that neither masking, nor duration were the causes of null findings in the aforementioned trials. Similarly, a lack of efficacy was apparent in another meta-analysis [94], revealing that isoflavone supplementation did not affect PSA concentrations among men with localized PCa.

Few RCTs have also examined the efficacy of phytoestrogens on the tertiary prevention level (Table 4). Sulforaphane is a natural compound found in many cruciferous vegetables, considered today as a “clinically relevant” nutraceutical for chronic disease management [101]. On this basis, Cipolla [95] examined the effect of 60 mg sulforaphane ONS compared with placebo among men with increasing PSA concentrations after prostatectomy. The findings revealed that PSA doubling time (PSADT) was prolonged by 86% in the intervention arm compared with the placebo group (28.9 and 15.5 months, respectively). In parallel, PSA increases >20% at 6 months were more frequent in the placebo group (71.8%) than the intervention arm. When men at high-risk of PCa recurrence after prostatectomy were randomized to a soy protein or placebo supplement for 2 years, the incidence of biochemical recurrence was similar among groups [96].

On the primary prevention level (Table 4), high versus low soy diet was compared, failing to reveal significant differences in PSA concentrations among groups [100]. Similarly, when older men, at risk of PCa due to their age, adhering to a high-soy diet or placebo for a year [98] did not demonstrate any differences in the recorded increases in PSA. In a phase II cross-over trial, Urban [97] randomized older men with high PSA concentrations to soy protein beverages with high or low genistein and daidzein content, however, no effect was noted on the PSA concentrations between intervention groups.

Resveratrol is a stilbene with antioxidant, estrogenic/antiestrogenic antitumor, and anti-carcinogenic properties [102,103] postulated mainly on the basis of in vitro studies [104]. Kjaer and associates [99] examined the effects of high vs. low doses of resveratrol in men with metabolic syndrome, however, no differences were noted in the concentrations of PSA, circulating testosterone, or the size of the prostate gland post-supplementation, between groups.

### 3.5. Green Tea Catechins

Tea, a common drink from the leaves of *Camellia sinensis*, is rich in polyphenols, including (−)-epigallocatechin-3-gallate (EGCG), (−)-epigallocatechin (EGC), (−)-epicatechin-3-gallate (ECG), and (−)-epicatechin (EC) [105]. Experimental trials have suggested that green tea catechins (GTCs) have inhibitory effects on carcinogenesis and cancer cell growth [105]. According to a recent Cochrane meta-analysis, the relative risk for developing PCa is reduced among green tea consumers [106]. Overall, GTCs appear to affect multiple cancer signaling pathways, and many RCTs have evaluated their effects in vivo (Table 5).

Among men with PCa, daily consumption of green tea on the weeks preceding prostatectomy induced a significant reduction in PSA compared with black tea, or water drinkers [107]. Unfortunately, these findings were not verified by further research. In a similar, but smaller trial, Wang et al. failed to report any PSA findings, although concentrations of serum PSA were also assessed [109]. When catechins supplementation was examined, it was not efficient in inducing differences in PSA concentrations of men scheduled for radical prostatectomy compared with controls [75]. Nevertheless, irrespectively on the PSA response to GTC supplementation, it has been suggested [113] that among men on active surveillance (AS), GTCs might improve quality of life and reduce overtreatment-related morbidities.

When patients with high-grade prostatic intraepithelial neoplasia (HGPIN) and/or atypical small acinar proliferation (ASAP) were recruited (Table 5), daily GTCs supplementation for a year decreased serum PSA concentrations compared with placebo, in two trials [110,112], but failed to induce any notable change according to one RCT [111]. In trials reporting a PSA decline however, the incidence of PCa did not differ among men receiving GTCs, or placebo [112].

### 3.6. Fruit (Cranberries, Pomegranate, Saw Palmetto, and Grapes) and Fruit Extracts

Fruits are rich sources of phytochemicals carrying multiple antioxidant and anti-inflammatory properties, and for this, a diet rich in fruits is often recruited to reduce oxidative stress in conditions with an elevated inflammatory state [114]. In parallel, fruit extracts derived usually via exploitation of fruit residues are often used in phytomedicine as nutraceuticals [115] with postulated cancer-related outcomes [116]. In BPH and PCa, in particular, apart from antioxidant and anti-inflammatory properties, fruits and fruit extracts are also expected to exhibit antiproliferative, anti-androgenic, and anti-estrogenic properties [117].

Cranberries (*Vaccinium macrocarpon*) are rich in bioactive compounds [118], including organic and phenolic acids, flavonoids and flavonoid glycosides, ursane triterpenoids, anthocyanins and proanthocyanidins [119], with many preclinical studies advocating for their effectiveness as an adjuvant PCa therapy [120]. One RCT [121] (Table 6) examined the effects of daily cranberry powder ONS compared with placebo on men with PCa, revealing a 22.5% reduction of PSA concentrations in the cranberry intervention arm after 30 days of supplementation. As far as PCa risk is concerned, another Czech RCT evaluated the efficacy of cranberry powder ONS—without a comparator—, reporting a reduction in PSA after 6 months of daily intake among men with lower urinary tract symptoms (LUTS), elevated PSA, but negative prostate biopsy [122].

According to the pharmaceutical industry, *Serenoa repens*, an extract from the saw palmetto fruit, appears to be a promising plant for PCa, with many RCTs examining its efficacy. In vivo studies postulate a reduction in inflammation, prostatic cell growth, and stimulation of the apoptotic machinery following saw palmetto ONS. RCTs on patients with BPH (Table 6) indicate that saw palmetto supplementation for 6 to 18 months failed to reduce PSA concentrations compared with placebo [123,124,125] or no comparator. The lack of significant findings was apparent even in cases where saw palmetto ONS was complemented with other herbal plants (nettle root, pumpkin seed oil) [126]. The Complementary and Alternative Medicine for Urologic Symptoms (CAMUS) trial [123,124] failed to record differences in PSA concentrations among men receiving escalating doses of saw palmetto extract compared with placebo. When saw palmetto was compared with effective medicine remedies like 5-alpha-reductase inhibitors, finasteride intake reduced PSA concentrations, whereas *Serenoa repens* ONS failed to induce any changes [127]. Similarly, compared with tamsulosin [125,128,129], saw palmetto did not have a significant effect on PSA concentrations after 6 [128] and 12 [130] months of supplementation. Moreover, according to a recent Chinese meta-analysis [131], saw palmetto was more effective in reducing PSA concentrations among men with BPH as compared with tamsulosin; however, in this analysis, non-randomized trials were analyzed together with RCTs. Moreover, combined tamsulosin and saw palmetto therapy were also inefficient in reducing PSA compared with tamsulosin alone [129]. However, based on yet another Chinese meta-analysis [132], when α-receptor blocker (αRB) monotherapy was compared with αRB, complemented by *Serenoa repens* extract intake, a significant decrease in PSA concentrations was noted among patients who were receiving the saw palmetto extract compared with αRB monotherapy, indicating a possible synergistic relationship.

Grapes (*Vitis vinifera*) and grape juice have also been studied in relation to PCa, due to the major phenolic antioxidants content (in grape skin and seeds extract) [138]. In the only double-blind published RCT to date (Table 6), Spettel et al. [133] compared the effects of the daily intake of 240 mL 100% Concord grape juice with placebo for a total of 3 months. The trial failed to report changes in PSA concentrations between the two interventions.

Pomegranate juice extract (POMx) is another popular nutraceutical with postulated anticancer effects due to its polyphenol-rich content. Among men with a histologic diagnosis of PCa, daily POMx supplementation did not reduce PSA concentrations before prostatectomy in a placebo-comparator RCT [134]. A similar lack of significant effect was also noted among men with rising PSA concentrations after primary therapy for PCa, who received 8 oz liquid POMx, or matching liquid placebo every day, for a year [136]. In a Swiss trial [135], the lack of efficacy of pomegranate juice in reducing PSA concentrations was also noted among men with histologically confirmed PCa. When high doses of POMx were compared with lower ones among participants with a rising PSA lacking without metastases, an overall increment in PSADT was noted [137].

Despite their apparent ineffectiveness, one common denominator in all fruit and fruit extract studies involves the adverse gastrointestinal events associated with high fruit intake [136,137], including nausea, constipation, diarrhea and decreased appetite, reducing compliance rate, and increasing the risk of dropping out from the trial.

### 3.7. Vitamin D

Vitamin D is an essential nutrient exerting a pleiotropic role in health attainment. Although some consider the vitamin as an antioxidant, high-quality evidence from human studies raised controversy concerning its ability to ameliorate oxidative stress in vivo [139]. Nevertheless, the role of vitamin D compounds in the treatment and causation of cancer has been examined and postulated since the 1970s [140]. The anti-tumor effect of the vitamin involves cell cycle arrest, inhibition of tumor cell proliferation, growth and invasiveness, and inflammatory signaling [141].

Although some cross-sectional, nested case-control or retrospective studies examining associations between serum 25-hydroxyvitamin D (25(OH)D) concentrations and PSA indicate the existence of an inverse relationship [142], not all of the published evidence is in agreement [141,143,144]. Nevertheless, in specific cancers, serum vitamin D_3_ concentrations are used as proxy biomarkers, with higher concentrations being indicative of a greater therapy response [145]. In parallel, a lack of association between serum 25(OH)D concentrations and PCa risk is also apparent [146,147].

Wagner et al. [148] compared escalating doses of vitamin D_3_ on patients with PCa before radical prostatectomy (Table 7). According to their findings, mega-doses of vitamin D_3_ (≥10,000 IU) reduced PSA concentrations compared with “normal” doses, not exceeding the recommended allowance ranges. Attia et al. [149] examined the effect of 1-alpha-hydroxy-vitamin D_2_ (doxercalciferol) in combination with intravenous (IV) docetaxel cycles on chemotherapy-naïve men with metastatic androgen-independent prostate cancer (AIPCa); however, no difference was noted in the circulating PSA concentrations post-intervention. In a similar trial, Beer [150] examined the combined effect of the active vitamin D form, calcitriol, and IV docetaxel, compared with IV docetaxel plus placebo, on men with progressive metastatic AIPCa. Once again, no differences were noted in the PSA response, or the duration of PSA progression-free survival between the two groups. When vitamin D_2_ ONS was compared with placebo in men with PCa scheduled for prostatectomy [151], a transient reduction was noted in PSA among the intervention group on day 21; however, this observation was later diminished before the end of the trial, which lasted for a total of 28 days. Moreover, the expression of PSA in adenocarcinoma did not differ between intervention and placebo groups [151]. Based on the aforementioned trials and the findings of a relevant systematic review by Petrou and associates [152], most RCTs suggested a lack of efficacy of vitamin D ONS in reducing PSA concentrations among men harboring PCa. These findings were also corroborated by a more recent meta-analysis, indicating a non-significant difference in PSA change, PSA response proportion and mortality rate between men with PCa receiving vitamin D, as compared with those randomized to a placebo [153]. 

Nevertheless, meta-analyses indicate that as far as PCa-mortality is concerned, increased serum 25(OH)D concentrations are associated with reduced mortality risk among patients with PCa [157], suggesting that the mediating effect of vitamin D extends beyond the circulating PSA concentrations. More recently, based on the androgen hypothesis [158], it was suggested that testosterone might modulate the 25(OH)D-PCa association [159], and, by inference, PSA concentrations.

In the primary prevention level (Table 7), vitamin D analogs ONS failed to induce significant changes in PSA concentrations among men with BPH compared with placebo [154,155,156], indicating a possible lack of efficacy.

### 3.8. Ascorbic Acid and A-Tocopherol

In a very early trial, Lasalvia-Prisco et al. [160] (Table 8) demonstrated that combined short-term ascorbic acid–menadione therapy produced an immediate drop in both tumor cell numbers and PSA concentrations among men with PCa, suggesting that autoschizis (cancer cell death characterized by a reduction in cell size that occurs due to the loss of cytoplasm through self-excision) can also be induced. However, the sample used was very small (*n* = 5 patients in each arm), and no information concerning blinding was reported.

The PREVENT trial [161] compared 400 IU vitamin E ONS with placebo among men with elevated PSA and/or abnormal digital rectal examination (DRE) on the baseline evaluation. Tocopherol supplementation for 18 months did not alter PSA concentrations. According to the Selenium and Vitamin E Cancer Prevention Trial (SELECT) however [61], comparing selenium to vitamin E, a combination of both, or placebo, longitudinal vitamin E ONS increased PCa risk by 17% within 7 years, without inducing significant changes in PSA concentrations, leaving a “bitter aftertaste” for the efficacy of the vitamin and terminating all relevant vitamin E-PCa therapies, due to safety concerns [162].

### 3.9. Combined Antioxidant Therapy

Given that individual antioxidants often failed to produce significant results in PSA concentrations among patients with PCa and or increased PCa risk, a combination of antioxidants is often used to tamper down PSA concentrations.

When men at risk for PCa were recruited (Table 9), high lycopene sources induced similar effects to PSA as soy-rich foods [163]. Similarly, no differences were noted when men with suspected PCa consumed saw palmetto, selenium, and lycopene or tamsulosin for a year according to the PROCOMB trial [164]. In HGPIN, supplementation with tomato products did not alter PSA compared with soy [165]. Similarly, a 6-month combined supplementation of lycopene, selenium, and GTCs among patients with primary multifocal HGPIN and/or ASAP was also ineffective concerning PSA kinetics [166]. A comparison of green tea against placebo, lycopene-rich foods, or lycopene capsules was also inefficient in down-regulating PSA [167]. However, in a similar trial where saw palmetto, selenium, and lycopene ONS was compared with no intervention, a reduction in PSA concentrations was reported after 6 months of supplementation [168]. Combined ONS of saw palmetto, quercetin, and β-sitosterol was also inefficient in reducing PSA concentrations [169]. In a placebo-controlled trial, curcumin and isoflavones synergistically reduced PSA concentrations among men who underwent systematic prostate biopsy due to elevated circulating PSA [170]. In BPH, cernitin, saw palmetto, B-sitosterol, and vitamin E combined therapy was ineffective in altering circulating PSA [171]. Similarly, saw palmetto and other herbs (pumpkin seed oil, nettle root) ONS failed to reduce PSA concentrations compared with placebo [126]. On the other hand, in combined LUTS and BPH, supplementation with selenium and silymarin for 6 months produced a significant reduction in PSA in the intervention group [172]. When apparently healthy men were recruited, a publication of the preliminary results of the SELECT trial [61,173] revealed that PSA concentrations and PSA velocity were indifferent among men receiving selenium, vitamin E, both, or placebo. Moreover, selenium ONS did not affect PCa incidence; however, vitamin E supplementation was associated with an increased PCa risk, leading to the termination of the trial for ethical reasons and subsequent continuation of the study in an observational manner [174]. Finally, ONS with combined vitamin C, α-tocopherol, β-carotene, selenium, and zinc did not affect PSA concentrations according to the SU.VI.MAX trial [175]. Therefore, it appears that with the exception of 3 trials [168,170,172], mixed antioxidant therapy does not appear to affect circulating PSA on the primary prevention level. Moreover, as indicated by few trials [61,65,176], antioxidant ONS might increase PSA and the risk for PCa, especially when baseline serum concentrations are high, and caution should be taken before consuming non-prescribed antioxidant supplements.

In the secondary prevention level (Table 9), as for lycopene-combined therapies, men with PCa following their usual diet, receiving tomato products containing 30 mg of lycopene, or tomato products plus selenium, n-3 fatty acids, soy isoflavones, grape/pomegranate juice, and green/black tea every day, failed to experience changes in PSA concentrations after 3 weeks of intervention [177]. Grainger [178] compared lycopene with soy supplementation for 8 weeks among 41 men with PCa, and although a significant reduction in PSA concentrations was reported in both arms, the crude significance level was not presented in the manuscript. In a similar population, supplementation with soy protein, vitamin E, and selenium for 3 years did not alter PSA concentrations compared with the placebo [165]. Similarly, ONS with vitamin E (350 mg), selenium (200 μg), vitamin C (750 mg), and coenzyme Q10 (CoQ10) (200 mg) for 21 weeks did not affect circulating PSA among men with hormonally untreated PCa and rising serum PSA concentrations [179]. On the other hand, mixed supplementation with broccoli powder, curcumin, pomegranate, and green tea extract, induced a lower rise in PSA in the intervention compared with the placebo group, among men with localized PCa after 6 months [180]. When men with PCa on AS were used, a combination of genistein, daidzein, and other isoflavones [181] failed to reduce PSA concentrations. In a phase II, cross-over trial, Oh et al. [182] examined supplementation with a mixed antioxidant containing medicinal mushroom extracts, *Seronoa repens*, and other substances against diethylstilbestrol, among men with histologic evidence of progressive androgen-independent PCa. Despite the exact duration of the trial not being reported, nor were differences in the PSA concentrations among participating groups. When a mixed antioxidant supplementation (including vitamin E, GTC, isoflavones, lycopene) was compared with placebo among men with hormonally untreated PCa and increasing PSA concentrations, total PSADT was unaffected and akin between groups [183]. Therefore, except for two trials [178,180], combined antioxidant supplementation appears ineffective in tampering down PSA concentrations among men with PCa. Along these lines, in a systematic review by Posadzki [184], the lack of efficacy of mixed antioxidant supplementation in reducing PSA concentrations among men with PCa was evident.

Silymarin is a milk thistle flavonolignan mixture [186], examined in several PCa-RCTs. In a tertiary prevention placebo-controlled trial, Schröder et al. [188] (Table 9) demonstrated that supplementation with soy, isoflavones, lycopene, silymarin, and antioxidants increased PSA doubling time by 2.6 (from 445 to 1150 days) among men with a history of PCa and rising PSA post-radical prostatectomy or radiotherapy. However, crude PSA concentrations post-intervention were not reported. Similarly, combined supplementation with silymarin and selenium failed to induce changes in PSA compared with placebo [186]. When the combined ONS included curcumin, resveratrol, GTC, and broccoli, no effect in PSA kinetics was noted among men with biochemically recurrent PCa and a moderate rise rate PSA, compared with placebo [187].

For many centuries, medicinal mushrooms were used in traditional medicine, with postulated efficacy stemming from direct tumor attack capacities, indirect defense, and T helper cell (Th) 1 immune response [189]. Yoshimura et al. [185] examined the efficacy of mushroom immunology in an oncology model, using men with biochemical failure after radical treatment for non-metastasized PCa. ONS with extracts from the *Agaricus blazei Murill* or the *Ganoderma lucidum* mushroom did not induce a different PSA response among men with biochemical failure after radical treatment for non-metastasized PCa [185]. However, a placebo-control group was not employed. 

Overall, based on the available data to date, mixed antioxidant therapy does not influence PSA concentrations on the primary, secondary, or tertiary prevention levels. Moreover, a lack of efficacy is also noted in RCTs with gene-expression outcomes, indicating that even in the genetic level, the impact of mixed antioxidant ONS is not justified [190]. In parallel, as already mentioned prior, antioxidant supplementation should not be unattended, or unprescribed. Several antioxidants are toxic at intake levels exceeding the recommended daily allowance (RDA), and this toxicity might reverse any efficacy, lead to poisoning (for some substances) and snowball adverse health events.

## 4. Fatty Acids (FA) and Foods Rich in Fatty Acids

Observational cohort studies indicate that the dietary intake of eicosapentaenoic (EPA; 20:5n−3), docosahexaenoic (DHA; 22:6n−3) may decrease the risk of PCa [191], whereas, according to animal studies, the intake of long-chain n-6 fatty acids enhances prostate tumor cell growth [192]. On the other hand, a decrease in the fatty acid content of the peri-prostatic adipose tissue has been observed with PCa aggressiveness [193]. Nevertheless, despite the ample research conducted on the subject, a salient explanation of the effect of FA intake on PCa is yet expected [194,195]. Aucoin et al. conducted a systematic review examining the effects of marine fatty acid supplementation, in particular, on serum PSA concentrations and PCa risk [23]. Based on the RCTs they included, intake of fish oil had no impact on PSA concentrations among patients with PCa, although some RCTs reported a decrease in inflammatory or other cancer markers [23]. In parallel, synthesis of cohort and case-control studies suggested an association between higher fish intake and deceased risk of PCa morality [23].

Among men with a PCa diagnosis (Table 10), based on the Molecular Effects of Nutritional Supplements (MENS) trial [196], three months of EPA and DHA supplementation did not induce changes in circulating PSA concentrations, compared with lycopene ONS. Similarly, in a Japanese trial without a comparator intervention, 2 years of EPA ONS failed to reduce PSA among men with PCa and low post-surgical PSA concentrations [197].

On the primary prevention level (Table 10), an 8-week walnut supplementation (75 g/d) diet among men at risk for PCa did not reduce PSA concentrations, although the ratio of free:total PSA was increased based on a non-predefined regression analysis [198]. Similarly, a cross-over, 6-month lower-dose walnut supplementation (35 g/d) with a 4-week washout interval, failed to reduce PSA among middle-aged men [199]. Similarly, the Alpha Omega trial [200] failed to demonstrate differences in the PSA concentrations of older adults with a history of myocardial infarction following 40 months of margarine spread-based α-linolenic acid (ALA) supplementation compared with placebo. When men were randomized to receiving EPA (2.4 g/d) for 12 weeks, or no intervention in the “Study of EPA Effects on Prostate Cancer” (SEEPC) [201], no differences in PSA response were recorded. Only one Iranian trial [202], suggested a reduction in PSA after 12 weeks of combined EPA and DHA supplementation, compared with placebo; however, participants were apparently healthy, without PCa-specific risk factors. Interestingly, in the same trial, parallel ONS with γ-linolenic acid increased circulating PSA among participants, indicating that not all fatty acids are effective, or risk-free.

In summary, based on the available RCTs, supplementation with fatty acids or foods rich in fatty acids does not appear to affect PSA concentrations among men with PCa or increased PCa risk.

## 5. Dietary Interventions

The search for the ideal diet delaying the progression of PCa and aiding towards therapy has been long. More than 20 years ago, for some researchers, PCa was considered a nutritional disease with its etiology stemming mainly from environmental factors [203]. This led to a plethora of “healthy” dietary patterns being studied concerning their anti-cancerogenic efficacy. However, according to a recent meta-analysis of cohort studies, adherence to traditionally considered “healthy” dietary models as the Mediterranean diet did not affect the incidence of PCa [204]. In contrast, the prostate testing for cancer and treatment (ProtecT) trial [205] showed that adherence to the PCa-specific dietary recommendations issued by the World Cancer Research Fund (WCRF) and the American Institute for Cancer Research (AICR) was associated with decreased risk of PCa and lower PSA concentrations.

Among men with PCa, several RCTs have been conducted, investigating the effects of distinct dietary patterns in reducing PSA concentrations (Table 11). For many years, meat and dairy products were considered as possible etiological factors of cancer. On this basis, Hébert et al. [206] randomized men treated by either prostatectomy, or radiation therapy to a healthy, low meat/dairy diet and increased physical activity intervention, or usual treatment. Adherence to the diet did not affect PSA concentrations at 6 months post-intervention. In support of their findings, a recent meta-analysis of cohort studies revealed that reduced meat intake is associated with a very small reduction in cancer risk, based mainly on evidence of low certainty [207]. 

In another trial, Demark-Wahnefried [208] decided to compare the efficacy of a low-fat diet (LFD) against the anti-inflammatory and anti-oncotic role of flaxseed lignans [209]. He assumed that a low-fat diet (LFD, <20% TEI), a high-flaxseed diet, or an LFD supplemented with flaxseed might entail health benefits for men with PCa scheduled for prostatectomy, compared with their usual diet [208]. Nevertheless, adherence to the three dietary interventions revealed a lack of statistical difference in circulating PSA between arms, at the time of prostatectomy [208]. Ornish [210] examined the efficacy of an intensive lifestyle intervention supporting an LFD supplemented with soy, fish oil, and antioxidant vitamins, with combined physical exercise and stress management among men with early, biopsy-proven PCa, against usual treatment. One year after the initiation of the trial, a favorable reduction was noted in the PSA concentrations of the intervention group. In the Men’s Eating and Living (MEAL) [211] study, telephone counseling promoting an increased vegetable intake for a total of two years failed to change PSA concentrations of men with biopsy-proven prostate adenocarcinoma (stage cT2a or less) and serum PSA concentrations not exceeding 10 ng/mL [211]. In parallel, time to progression (TTP), defined as PSA exceeding 10 ng/mL, did not differ between the participating groups. Aronson et al. [212] compared the typical Western diet to a low fat/fish oil (LFFO) diet among men scheduled for radical prostatectomy. After 4–6 weeks (from initiation of the intervention, until the day of the operation), the PSA concentrations of the two intervention arms remained indifferent. In a subsequent trial, Aronson et al. [213] examined the effect of a high-fiber LFD against the typical Western diet among men who had not received prior therapy for PCa. A lack of significance was noted once more, concerning PSA concentrations of participants, 4 weeks after the trial.

Approximately 100 years ago, Otto Warburg noted that tumors demonstrate a tendency to metabolize glucose anaerobically, even when oxygen availability is adequate [214]. To reverse this phenomenon and subsequently, inhibit tumor growth in a multicellular environment, the intake of low carbohydrate diets was proposed as a hypothesis, based mainly on results from animal studies [215,216]. In the carbohydrate and prostate study 1 (CAPS1) [217], Freedland et al. decided to examine the Warburg effect [214] on men with PCa on androgen deprivation therapy. Participants either reduced the carbohydrate content of their diets and increased their physical activity level, or retained their usual diet, for 6 months. No differences were exhibited in the PSA concentrations of participants in the intervention, or usual diet arms. Similar findings were also reported at the Carbohydrate and Prostate Study 2 (CAPS2) [218]. The low carbohydrate content of the diets induced mild adverse events, including constipation and fatigue; however, all were reported to be diminished by the end of the 6-month intervention.

In the tertiary prevention level (Table 11), Li et al. [219] tested the efficacy of an LFD, with a high fiber content, supplemented with soy and supported by individual counseling sessions among men at high risk for PCa recurrence, after prostatectomy. Four years of adherence did not reduce PSA concentrations compared with the typical US Department of Agriculture (USDA) diet. Similarly, delivery of dietary and cooking classes for 3 months to men previously treated for PCa and their partners did not alter the rate of participants with increased PSA concentrations compared with no intervention, but effectively prolonged PSADT in the arm receiving the counseling [220]. When men with a history of PCa and rising PSA concentrations after primary therapy (prostatectomy or radiation) were randomized to a combined intervention including dietary modifications, increased physical activity and stress-reduction or standard care, no differences were noted in the PSA concentrations between groups [142].

When men at risk for PCa were recruited, the Prostate Cancer Prevention Trial (PCPT) [221] failed to reveal differences in the PSA concentrations of men receiving intensive counseling for an LFD, high in fiber, fruits, and vegetables for two years, compared with those receiving the standard brochure on what is considered as a “healthy” diet. In parallel, the incidence of PCa in four years was similar in both arms [221]. Similarly, the Polyp Prevention Trial (PPT) [222] did not indicate differences in the PSA kinetics of men adhering to a LFD, high in fiber, fruits, and vegetables, compared with those maintaining their usual diet for a total of four years. Although, ethnic differences were noted in the incidence of PCa in the sample, these were not related to the fat content of the diet, followed by the participants [222]. Tariq and associates [223] compared the effect of adhering to diets high in soluble fiber or diets high in insoluble fiber, on the PSA concentrations of men with hyperlipidemia. After 4 months of intervention, serum PSA concentrations were lower in the soluble fiber groups as compared to the insoluble fiber, but no treatment differences were observed in serum sex hormones concentrations (free testosterone or estradiol) [223]. According to the authors, soluble fiber increased fecal steroid elimination, leading to a reduction in PSA concentrations [223,224].

## 6. Lack of Efficacy, Drawbacks in the Amount of PSA Considered as “Elevated”, and Food for Thought

Considering that the prostate gland has a very efficient blood supply [6], amounts of PSA are constantly entering the bloodstream, elevating circulating concentrations. This often produces concentrations exceeding the 3 ng/mL, which is considered the cutoff for PCa risk, developing a grey area in what is considered as “normal” and what is thought to be “elevated” [6]. Elevated PSA concentrations might well be the epiphenomenon of urinary tract infections, prostatitis, indirect pressure on the prostate gland, or BPH. Therefore, these factors, alongside age, ethnicity, and BMI, might account for the reported inconsistency in the findings of all aforementioned trials and should all be considered as confounders when relevant RCTs are designed.

Furthermore, the inconsistency of the findings reported in the present review is also greatly dependent on masking, degree of intervention adherence, variability of circumstances, and sample size adequacy. In parallel, nutrition interventions carry more limitations as compared to drug RCTs [225], ailing nutrition research. It has been suggested that in the science of nutrition, even similar RCTs can yield different results, indicating that often the postulated effect is most likely, inexistent [226]. Moreover, the need for incorporating personalized nutrition in nutritional interventions is also important, as response to supplementation is often genotype-dependent [227].

Nevertheless, often, men with PCa exhibit low PSA concentrations [228], whereas the majority of the evidence concerning PSA is based on studies conducted in men aged between 55 to 69 years [1]. Despite its limitations, PSA is the only relevant marker available at the moment and a common outcome in all PCa-related research.

On the other hand, it is not known whether a reduction in circulating PSA concentrations decreases the risk of PCa diagnosis or just masks the risk of PCa. Although some studies included herein suggested a reduction in PCa risk using diagnosis, recurrence, or PCa-specific mortality as outcomes, not all RCTs have reported relevant findings. Subsequently, caution is required when non-specific biomarkers are used as proxies for PCa in RCTs without the inclusion of PCa-specific outcomes. 

## 7. Conclusions

In the phytotherapeutic field, it appears that high-quality studies are lacking, with the majority of RCTs being underpowered [229]. In parallel, as already mentioned by others, PCa chemoprevention by natural agents is not supported by the available evidence [230]. Therefore, the need for well-designed trials to expand the existing knowledge, replicate the findings, and aggregate the results is necessitated [231]. Moreover, the efficacy of supplements and, in particular, antioxidants, in tampering down PSA concentrations is questionable and a “first do not harm” concept must prevail before the formulation of any recommendations [232]. Given that the majority of evidence outlined in the present review revealed little or no effect, it can be assumed that nutrition-wise, PSA is not as sensitive as we might think, whereas the role of nutrition in down-regulating PSA concentrations appears to be minimal.

## Figures and Tables

**Table 1:** RCTs examining the effect of lycopene ONS, or the consumption of tomato-products on the PSA concentrations among men with PCa, increased PCa risk, or BPH.

First Author	Origin	Masking	Duration	Patients	Interventions	Results
Ansari [47]	IN	NR	6 mo	*n* = 44 men with metastatic PCa (M1b or D2)	1. Orchidectomy (*n* = 27) 2. Orchidectomy + lycopene ONS started on the orchidectomy day (2 × 2 mg/d) (*n* = 27)	At 6 mo, a reduction in PSA was noted in both arms, but at 2 yrs, it was higher in the lycopene arm. More men on lycopene had a complete PSA response.
Kucuk [49]	US	NR	3 wks until prostatectomy	*n* = 26 men with PCa	1. Tomato extract (30 mg of lycopene) (*n* = 15) 2. No ONS (*n* = 11)	Subjects in the tomato arm had lower PSA.
Kumar [50]	US	Double-blind	From biopsy to prostatectomy (approx. 30 d)	*n* = 45 men with PCa, before prostatectomy	1. Lycopene (15 mg) (*n* = 10) 2. Lycopene (30 mg) (*n* = 10) 3. Lycopene (45 mg) (*n* = 14) 4. No ONS (*n* = 11)	No difference was noted in PSA concentrations between treatment arms.
Bunker [48]	US	Open-label	4 mo	*n* = 77 AC men with HGPIN, atypical foci or repeated non-PCa biopsies	1. Lycopene (30 mg) + MV (*n* = 38) 2. MV (*n* = 39)	PSA declined during the 1st mo but returned to baseline concentrations by mo 4. The PSA response was identical in both groups.
Schwarz [52]	DE	Double-blind	6 mo	*n* = 40 men with BPH, free of PCa	1. Lycopene (15 mg) (*n* = 20) 2. Placebo (*n* = 20)	Supplementation decreased PSA concentrations in the intervention group.

AC, Afro-Caribbean; BPH, benign prostate hyperplasia; HGPIN, high-grade prostatic intraepithelial neoplasia; MV, multivitamin; NR, not reported; ONS, oral nutrient supplement; PCa, prostate cancer; PSA, prostate-specific antigen; RCTs, randomized controlled trials.

**Table 2:** RCTs examining the effect of selenium ONS on the PSA concentrations among men with PCa or increased PCa risk.

First Author	Origin	Masking	Duration	Patients	Interventions	Results
Stratton ^†^ [65]	US	Double-blind	Up to 5 yrs	*n* = 140 men with localized non-metastatic PCa	1. Se (200 μg/d) (*n* = 47) 2. Se (800 μg/d) (*n* = 47) 3. Placebo (*n* = 46)	Adjustment for age, BMI, baseline Se and PSA, smoking, race, PSA method, and Gleason score, PSA velocities for the two treatment arms did not different from the placebo.
Algotar ^Š^ [60]	US	Double-blind	Up to 5 yrs	*n* = 699 men at high PCa risk (PSA > 4 ng/mL and/or suspicious DRE and/or PSA velocity >0.75 ng/mL/yr) and a negative biopsy	1. Se (200 µg) (*n* = 234) 2. Se (400 µg) (*n* = 233) 3. Placebo (*n* = 232)	PSA velocity in the Se arms did not differ from the placebo group.

BMI, body mass index; DRE, digital rectal examination; ONS, oral nutrient supplement; PCa, prostate cancer; PSA, prostate-specific antigen; RCT, randomized controlled trials; Se, selenium. ^†^ phase II trial; ^Š^ phase III trial.

**Table 3:** RCTs examining the effect of curcumin ONS on PSA concentrations among men with PCa.

First Author	Origin	Masking	Duration	Patients	Interventions	Results
Choi [76]	KR	Double-blind	6 mo	*n* = 97 patients with PCa receiving IAD	1. Oral curcumin (1.44 g/d) (*n* = 49) 2. Placebo (*n* = 48)	The % of patients with PSA progression was lower in the curcumin arm. PSA did not differ.
Hejazi [75]	IR	NR	3 mo	*n* = 40 patients with PCa on EBRT (<74 Gy)	1. Oral curcumin (3 g/d) (*n* = 20) 2. Placebo (*n* = 20)	PSA concentrations were reduced below 0.2 ng/mL in both groups.

EBRT, external beam radiation therapy; IAD, intermittent androgen deprivation; NR, not reported; ONS, oral nutrient supplement; PCa, prostate cancer; PSA, prostate-specific antigen; RCT, randomized controlled trials.

**Table 4:** RCTs examining the effect of phytoestrogen ONS on PSA concentrations among men with PCa, or increased PCa risk.

First Author	Origin	Masking	Duration	Patients	Interventions	Results
Lazarevic ^†^ [84,85]	NO	Double-blind	3–6 wks until prostatectomy	*n* = 47 men with localized PCa	1. 30 mg synthetic genistein (*n* = 24) 2. Placebo (*n* = 23)	Serum PSA was indifferent between groups. In the genistein arm, PSA in tumor and normal tissue were akin.
Dalais [89]	AU	Double-blind	Approx. ° 25 d	*n* = 26 men with PCa, scheduled to undergo radical prostatectomy	1. Bread with 50 g of HT soy grits (*n* = 8) 2. Bread with 50 g HT soy grits + 20 g linseed (*n* = 10) 3. Wheat bread (*n* = 8)	Differences were noted between the HT soy grits arm and the control (wheat) group on the % Δ total PSA and the % Δ in free/total PSA ratio.
Kumar ^†^ [90]	US	Double blind	3–6 wks to prostatectomy	*n* = 62 men with PCa	1. Aglycone isoflavones (40 mg) (*n* = 31) 2. Placebo (*n* = 31)	Change in PSA was not significant.
Kumar ^∞^ [91]	US	NR	from biopsy to prostatectomy (30 ± 3 d)	*n* = 44 men (45–80 yrs) with clinically localized PCa	1. Isoflavones (40 mg) (*n* = 12) 2. Isoflavones (60 mg) (*n* = 11) 3. Isoflavones (80 mg) (*n* = 10) 4. No intervention (*n* = 11)	Changes in serum PSA were not significant.
Hamilton-Reeves [92]	US	Double blind	up to 6 wks to prostatectomy	*n* = 86 men with localized PCa	1. Soy isoflavone capsules (80 mg/d of total isoflavones) (*n* = 42) 2. Placebo (*n* = 44)	Changes in serum PSA were not significant.
Kumar [93]	US	Double-blind	12 wk	*n* = 76 men with PCa, Gleason ≤6, (50–80 yrs)	1. Isoflavones (60 mg/d) (*n* = 39) 2. Placebo (*n* = 37)	No difference was noted in free or total PSA between groups.
Bylund [87]	SE	NR	3 wk	*n* = 16 men with conservatively treated PCa	1. 295 g of rye bran bread (*n* = 8) 2. 275 g of wheat bread (*n* = 8)	The changes in PSA were not significant.
Landberg ^‡^ [88]	SE	NR	6 wk each, 2 wk washout	*n* = 17 men with PCa	1. 485 g rye whole grain and bran products (50% TEI) (*n* = 9) 2. Refined wheat products with added cellulose (50% TEI) (*n* = 8)	Total PSA did not change from baseline, but it was lower at 2 wk in the rye whole grain arm.
Cipolla [95]	FR	Double-blind	8 mo	*n* = 77 men with high PSA post-prostatectomy	1. Mo 1–6: sulforaphane (60 mg); Mo 7–8: no ONS (*n* = 38) 2. Placebo (*n* = 39)	PSADT was 86% longer in the intervention group. PSA increases >20% (6 mo) were greater in the placebo (71.8%).
Bosland [96]	US	Double-blind	2 yr	*n* = 151 men at high-recurrence risk	1. Soy PRO (*n* = 78) 2. Placebo (*n* = 73)	28.3% of men developed biochemical recurrence within 2 yrs (NS).
Urban [97] ^†,‡^	US	Double-blind	6 wks	*n* = 34 older men with high PSA concentrations	1. Soy PRO beverages twice/d (with 42 mg genistein and 27 mg daidzein and other micronutrients) (*n* = 17) 2. Soy PRO beverages twice/d (2.1 mg genistein and 1.3 mg daidzein and other micronutrients) (*n* = 16)	The changes in PSA were not significant.
Adams [98]	US	Double-blind	12 mo	*n* = 150 healthy, older men	1. Soy PRO drink (83 mg/d isoflavones) (*n* = 74) 2. Drink without isoflavones (*n* = 76)	Serum PSA concentrations increased in both groups over the intervention, with changes being akin.
Kjaer [99]	DK	Double-blind	4 mo	*n* = 66 middle-aged men with MetS	1. Resveratrol (150 mg/d) 2. Resveratrol (1000 mg/d)	Prostate size and concentrations of PSA, testosterone, free testosterone and DHT remained unchanged.
Maskarinec ^‡^ [100]	US	Open-label	3 mo	*n* = 23 men NOD	1. High soy diet (*n* = 12) 2. Low soy diet (*n* = 11)	A 14% decline in serum PSA concentrations (NS) was observed with the high soy diet compared with the low.

Δ, change; DHT, dihydrotestosterone; HT, heat-treated; MetS, metabolic syndrome; NOD, not other defined; NS, not significant; ONS, oral nutrient supplement; PCa, prostate cancer; PRO, protein; PSA, prostate-specific antigen; PSADT, prostate-specific antigen doubling time; RCT, randomized controlled trials; TEI, total energy intake. ^∞^ phase I trial; ^†^ phase II trial; ^‡^ cross-over trial; ° exact mean duration was not reported.

**Table 5:** RCTs examining the effect of green tea and/or GTC supplementation on PSA concentrations among men with PCa or increased PCa risk.

First Author	Origin	Masking	Duration	Patients	Interventions	Results
Henning ^†^ [107]	US	Open-label	d-33, d-31, and d-29, respectively in each group	*n* = 93 men diagnosed with PCa before radical prostatectomy	1. 6 cups/d of brewed green tea (*n* = 34) 2. 6 cups/d of black tea (*n* = 26) 3. 6 cups/d of water (control) (*n* = 33)	A small decrease was noted in serum PSA concentrations among green tea drinkers, compared with the controls.
Nguyen [108]	US	Double-blind	3–6 wks until prostatectomy	*n* = 50 men with a PCa diagnosis scheduled for prostatectomy	1. PolyE ^∂^ (800 mg EGCG) (*n* = 25) 2. Placebo (*n* = 25)	No difference was noted in PSA concentrations between groups.
Wang [109]	US	NR	3–6 wks until prostatectomy	*n* = 17 men with clinically localized PCa	1. 6 cups green tea/d (*n* = 8) 2. 6 cups water/d (*n* = 9)	PSA results were not reported.
Kumar [110]	US	Double-blind	1 yr	*n* = 97 men with HGPIN and/or ASAP	1. PolyE ^∂^ (a mixture of GTCs with 400 mg EGCG)/d (*n* = 49) 2. Placebo (*n* = 48)	A decrease in serum PSA was observed on the PolyE arm.
Bettuzzi [111]	IT	Double-blind	1 yr	*n* = 60 men with HGPIN	1. 3 × 200 mg GTCs caps 2. Placebo	PSA did not change between the two arms.
Micali ^†^ [112]	IT	Double-blind	1 yr	*n* = 44 patients with HGPIN	1. GTCs (600 mg/d) (*n* = 22) 2. Placebo (*n* = 22)	A reduction was noted in PSA of the GTCs arm at 6 and 12 mo.

ASAP, atypical small acinar proliferation; CI, confidence intervals; EGCG, (-)-epigallocatechin-3-gallate; GTCs, green-tea catechins; HGPIN, high-grade prostatic intraepithelial neoplasia; PCa, prostate cancer; PolyE, polyphenon E; PSA, prostate-specific antigen; RCT, randomized controlled trials. ^†^ phase II trial; ^∂^ contains 85–95% total catechins, 56–72% as EGCG, and <1.0% caffeine.

**Table 6:** RCTs examining the effect of fruit and fruit extract supplementation on PSA concentrations among men with PCa, increased PCa risk, or BPH.

First Author	Origin	Masking	Duration	Patients	Interventions	Results
Student [121]	CZ	Double-blind	30 d	*n* = 64 men with PCa prior to surgery	1. Cranberry fruit powder (1.5 g) (*n* = 32) 2. Placebo powder (*n* = 32)	Serum PSA concentrations decreased by 22.5% in the cranberry intervention arm.
Vidlar [122]	CZ	NR	6 mo	*n* = 42 men at risk of PCa with LUTS, elevated PSA and negative biopsy	1. Dried powdered cranberries (0.5 g/d) (*n* = 21) 2. No cranberry treatment (*n* = 21)	The cranberry group experienced a reduction in PSA concentrations on d 180.
Spettel [133]	US	Double-blind	3 mo	*n* = 113 men (>45 yrs) with significant LUTS	1. Concord grape juice (240 mL/d) (*n* = 57) 2. Placebo (*n* = 56)	No statistical difference was observed between groups by PSA.
Freedland ^†^ [134]	US	Double-blind	4 wks	*n* = 63 men with PCa, scheduled for prostatectomy (>2 wks)	1. 2 x 2 POMx caps (each, 0.6 g polyphenols) (*n* = 30) 2. Placebo (*n* = 33)	No differences between arms in pre-surgical PSA or the ratio of baseline/pre-surgery PSA.
Stenner-Liewen [135]	CH	Double-blind	4 wks	*n* = 87 men with histologically confirmed PCa and PSA ≥ 5 ng/mL	1. Pomegranate juice 500 mL/d (*n* = 45) 2. Placebo beverage 500 mL/d (*n* = 42)	No differences were detected regarding PSA kinetics.
Pantuck [136]	US	Double-blind	12 mo	*N* = 166 men with rising PSA concentrations after primary PCa therapy	1. 8 oz liquid POMx (1.6 mmol polyphenols/d) (*n* = 102) 2. Matching liquid placebo (*n* = 64)	POMx did not prolong PSADT (crude PSA concentrations not compared).
Paller ^†^ [137]	US	Double-blind	18 mo	*n* = 100 men with a rising PSA, without metastases	1. POMx (1 g/d) (*n* = 50) 2. POMx (3 g/d) (*n* = 50)	POMx was associated with ≥6 mo higher PSADT (no crude PSA concentrations reported).
Ryu [129]	KR	Open-label	1 yr	*n* = 120 men with symptomatic BPH	1. Tamsulosin (0.2 mg/d) + saw palmetto (320 mg/d) (*n* = 60) 2. Tamsulosin (0.2 mg/d) only (*n* = 60)	No differences were noted in PSA concentrations among patients between groups.
Barry [123,124]	US	Double-blind	72 wks	*n* = 357 men (>45 yrs) with an AUA symptom score of 8–24	1. Saw palmetto (320 mg, wks 0–24; 640 mg, wks 24–48; 960 mg, wks 48–72) (*n* = 176) 2. Placebo (*n* = 181)	No difference was recorded in the PSA concentrations between groups.
Bent [125]	US	Double-blind	1 yr	*N* = 225 men (>49 yrs) with mild-to-severe BPH symptoms	1. Saw palmetto extract (2 × 160 mg/d) (*n* = 112) 2. Placebo (*n* = 113)	No difference in the PSA concentrations between groups.
Debruyne [130]	MC	Double-blind	12 mo	*n* = 704 men with symptomatic BPH	1. Tamsulosin (0.4 mg/d) (*n* = 354) 2. Saw palmetto (320 mg/d) (*n* = 350)	PSA remained stable without differences between groups.
Carraro [127]	FR	Double-blind	6 mo	*n* = 951 men with moderate BPH	1. Saw palmetto extract (320 mg) (*n* = 467) 2. Finasteride (5 mg) (*n* = 484)	PSA concentrations fell after 13 wks of finasteride but remained stable with saw palmetto.
Argirović [128]	RS	NR	6 mo	*n* = 265 men with LUTS due to BPH	1. Tamsulosin (0.4 mg) (*n* = 87) 2. saw palmetto (320 mg) (*n* = 97) 3. Tamsulosin (0.4 mg) + saw palmetto (320 mg) (*n* = 81)	No differences in the PSA concentrations were recorded between groups.

AUA, American Urological Association; BPH, benign prostate hyperplasia; eq, equivalent; LUTS, lower urinary tract symptoms; mc, multi-country; PCa, prostate cancer; POMx, pomegranate extract; PSA, prostate-specific antigen; PSADT, prostate-specific antigen doubling time; RCT, randomized controlled trials; ^†^ phase II trial.

**Table 7:** RCTs examining the effect of vitamin D ONS on PSA concentrations among men with PCa or increased PCa risk.

First Author	Origin	Masking	Duration	Patients	Interventions	Results
Wagner [148]	CA	Double-blind	3–8 wks	*n* = 65 patients with PCa (Gleason 6 or 7), before radical prostatectomy	1. 400 IU vitamin D_3_ (*n* = 20) 2. 10,000 IU vitamin D_3_ (*n* = 22) 3. 40,000 IU vitamin D_3_ (*n* = 23)	Serum PSA was lower in the combined higher-dose groups (10,000 and 40,000 IU) at the end of the trial.
Gee ^†^ [151]	US	Open-label	28 d	*n* = 31 clinically organ-confined PCa and HGPIN, scheduled for RRP	1. 10 μg vitamin D_2_ (*n* = 16) 2. Placebo (*n* = 15)	PSA was significantly lower on d-21 in the intervention, but indifferent at the LOCF. The expression of PSA in adenocarcinoma did not differ between groups.
Attia ^†^ [149]	US	Double-blind	17.6 mo	*n* = 70 Chemotherapy-naive men with metastatic AIPCa	1. IV docetaxel (35 mg/m^2^ (days 1, 8, 15) + doxercalciferol ONS (10 mg, days 1–28) (*n* = 37) 2. IV docetaxel (35 mg/m^2^ (days 1, 8, 15) + placebo (*n* = 33)	No difference in the PSA response rate was noted between groups.
Beer [150]	US	Double-blind	6 mo	*n* = 250 men with progressive metastatic AIPCa and adequate organ function	1. IV docetaxel (36 mg/m^2^/wk) for 3 wks of a 4-wk cycle + 45 mg calcitriol taken 1 d before docetaxel (*n* = 125) 2. IV docetaxel (36 mg/m^2^/wk) for 3 wks of a 4-wk cycle + placebo ONS taken 1 d before docetaxel (*n* = 125)	PSA responses were observed in 58% of calcitriol patients and 49% of placebo patients (NS). The median duration of PSA progression-free survival was 7.6 mo in the placebo group and 7.9 mo among calcitriol-treated patients.
Safwat [154]	EG	Open-label	2 yrs	*n* = 389 naïve BPH patients with moderate/severe symptoms	1. Tamsulosin (*n* = 193) 2. Tamsulosin + vitamin D_3_ (*n* = 196)	Patients receiving vitamin D_3_ had reduced PSA concentrations at the end of the treatment period (0.27 ± 0.08 ng/mL).
Chandler [155]	US	Double-blind	3 mo	*n* = 105 Black men NOD	1. Placebo (*n* = 27) 2. 1000 IU vitamin D_3_ (*n* = 21) 3. 2000 IU vitamin D_3_ (*n* = 28) 4. 4000 IU vitamin D_3_ (*n* = 29)	No differences in free and total PSA were observed.
Colli [156]	IT	Double-blind	12 wks	*n* = 119 patients with BPH	1. Vitamin D_3_ analog (150 μg/d) (*n* = 57) 2. Placebo (*n* = 62)	The change in PSA concentrations between groups was not significant.

AIPCa, androgen-independent prostate cancer; BPH, benign prostatic hyperplasia; HGPIN, high grade prostatic intraepithelial neoplasia; IU, international units; IV, intravenous; LOCF, last-observation carry forward; NOD, not other defined; PCa, prostate cancer; PSA, prostate-specific antigen; RCT, randomized controlled trials; RRP, radical retropubic prostatectomy. ^†^ phase II trial.

**Table 8:** RCTs examining the effect of vitamin C, or α-tocopherol ONS on PSA concentrations among men with PCa or increased PCa risk.

First Author	Origin	Masking	Duration	Patients	Interventions	Results
Lasalvia-Prisco [160]	IT	NR	42 d	*n* = 20 men with PCa	1. Vitamin C (5 g/m^2^/d) + menadione (50 mg/m^2^/d) on 7-d courses, beginning on d-1 and 22 (*n* = 5) 2. Menadione (50 mg/m^2^/d) on 7-d courses, beginning on d-1 and 22 (*n* = 5) 3. Vitamin C (5 g/m^2^/d) on 7-d courses, beginning on d-1 and 22 (*n* = 5) 4. placebo (*n* = 5)	For group 1, the rise of PSA at d-15 and the fall of PSA at d 22, 29, 36, and 42 were different compared with the controls.
Herná-andez [161]	US	Double-blind	18 mo	*n* = 44 men with high PSA and/or abnormal DRE	1. 400 IU vitamin E (*n* = 22) 2. Placebo (*n* = 22)	Tocopherol supplementation did not affect PSA.

DRE, digital rectal examination; IU, international units; PCa, prostate cancer; PSA, prostate-specific antigen; RCT, randomized controlled trials.

**Table 9:** RCTs examining the effect of combined antioxidant supplementation on PSA concentrations among men with PCa or increased PCa risk.

First Author	Origin	Masking	Duration	Patients	Interventions	Results
Paur [177]	NO	Single-blind	3 wks	*n* = 86 men with PCa	1. Tomato products (30 mg lycopene/d) (*n* = 28) 2. Tomato products + Se, n-3 fatty acids, soy isoflavones, grape/pomegranate juice, green/black tea (*n* = 28) 3. Control diet (*n* = 30)	No differences in the PSA concentrations between the intervention and control groups were noted.
Grainger [178]	US	NR	8 wks	*n* = 41 men with PCa	1. Wks 0–4: Tomato products (no soy) (>25 mg of lycopene/d). Wks 4–8: a combined tomato-rich diet and soy ONS (*n* = 20) 2. Wks 0–4: Soy (no tomatoes) (40 g of soy protein/d) Wks 4–8: a combined tomato-rich diet and soy ONS (*n* = 21)	A reduction in % PSA concentrations was noted in the tomato (25%) and soy (43%) groups without any statistics being presented between groups.
Hoenjet [179]	NL	Double-blind	21 wks	*n* = 70 patients with hormonally untreated PCa and rising PSA	1. vitamin E (350 mg), Se (200 μg), vitamin C (750 mg), CoQ10 (200 mg) (*n* = 36) 2. placebo (*n* = 34)	ONS with a combination of vitamin E, Se, vitamin C and CoQ10 did not affect serum PSA concentrations.
Oh ^†,‡^ [182]	US	NR	NR	*n* = 85 men with histologic evidence of progressive androgen-independent PCa	1. 3x3 PC-SPES caps with contained 320 mg of herbal combination (*Ganoderma lucidum*, *Scutellaria baicalensis*, *Rabdosia rubescens*, *Isatis indigotica*, *Dendranthema morifolium*, *Seronoa repens*, *Panax pseudoginseng*, and *Glycyrrhiza uralensis*) (*n* = 43) 2. DES (*n* = 42)	Among those treated with PC-SPES, 6/16 patients had decreases in PSA. Among those treated with DES, 2/8 patients had a decrease in PSA, though neither achieved a 50% PSA response.
Kranse ^‡^ [183]	NL	Double-blind	6 wk each arm with washout	*n* = 66 men with hormonally untreated PCa and increasing PSA concentrations	1. Margarine [with plant estrogens (1.5 g), Vitamin E (50 mg), Se (0.2 mg)], carotenoids (10 mg lutein, 10 mg lycopene, 10 mg palm carotenoids), green tea (6 cups), isoflavones (100 mg phytoestrogens, 60 mg genistein, 40 mg daidzein) (*n* = 15) 2. placebo (*n* = 16)	Total PSADT was unaffected. Free PSA increased during the placebo phase and decreased during the supplement period.
DeVere White [181]	US	Double-blind	6 mo	*n* = 53 men with PCa on AS	1. Genistein (450 mg), daidzein (300 mg), other isoflavones (*n* = 36) 2. Placebo (*n* = 30)	PSA concentrations did not change in either group after intervention.
Thomas [180]	GB	Double-blind	6 mo	*n* = 199 men with localized PCa	1. Broccoli powder (100 mg) + turmeric (100 mg) + pomegranate (100 mg) + green tea 5:1 extract (10 mg) (*n* = 134) 2. Placebo (*n* = 65)	A lower rise in PSA was observed in the supplement group, as opposed to the placebo.
Yoshimura [185]	JP	Open-label	6 mo	*n* = 47 men with biochemical failure after radical treatment for non-metastasized PCa	1. Senseiro (*Agaricus blazei Murill* mushroom) (*n* = 32) 2. Rokkaku Reishi (*Ganoderma lucidum* mushroom) (*n* = 15)	No partial response in terms of PSA was observed. At 12 mo after entry, the PSADT of the Senseiro group was not prolonged and that of the Rokkaku Reishi arm was marginally prolonged compared with baseline values.
Vidlal [186]	CZ	Double-blind	6 mo	*n* = 37 men, 2–3 mo post-radical prostatectomy	1. Silymarin (570 mg) + Se (240 μg) (*n* = 19) 2. Placebo (*n* = 18)	No difference in the PSA was noted between groups.
Van Die [187]	AU	Double-blind	12 wks	*n* = 20 men with biochemically recurrent PCa and a moderate rise rate PSA	1. 2 × 2 caps/daily containing curcumin (100 mg), resveratrol (120 mg), GTC (100 mg) + 2 × 2 caps broccoli (equivalent to 2 g fresh sprouts) (*n* = 9) 2. Placebo (*n* = 11)	The active treatment arm experienced a non-significant increase in the log-slope of PSA, and the placebo arm experienced no change in the log-slope of PSA.
Schröder ^‡^ [188]	NL	Double-blind	10 wks each, 4-wk washout	*n* = 49 men with PCa history and rising PSA post-radical prostatectomy or radiotherapy	1. Soy, isoflavones, lycopene, silymarin, antioxidants (*n* = 49) 2. Placebo (*n* = 49)	A 2.6-fold increase in PSADT from 445 to 1150 d was recorded for the supplement and placebo periods, respectively.
Gontero ^†^ [166]	IT	Double-blind	6 mo	*n* = 60 men with primary mHGPIN and/or ASAP	1. Lycopene (35 mg), Se (55 µg), and GTCs (600 mg) (*n* = 30) 2. placebo (*n* = 30)	No significant variations in PSA concentrations were observed.
Vostalova [172]	CZ	Double-blind	6 mo	*n* = 55 men with LUTS, BPH and PSA ≤ 2.5ng/mL	1. Se (240 μg) + silymarin (570 mg) (*n* = 26) 2. Placebo (*n* = 29)	A significant reduction in PSA in the intervention group was observed.
Fleshner [165]	US	Double-blind	3 yrs	*n* = 303 men with HGPIN	1. 2× soy protein (20 g), vitamin E (400 IU), Se (100 μg) 2. Whey-based placebo	No differences were recorded in the PSA concentrations of participants between the two arms.
Vaishampayan [163]	US	NR	6 mo	*n* = 71 patients with 3 successive rising PSA concentrations or >PSA of 10 ng/mL at 2 alternate evaluations	1. 2 × 1 tomato extract caps (15 mg of lycopene) (*n* = 38) 2. 2 × 1 tomato extract caps (15 mg of lycopene) plus 2 × 1 caps (40 mg soy isoflavone mixture) (*n* = 33)	No decline in serum PSA was noted in either group.
Lane ^†^ [167]	GB	Double-blind (caps), single-blind (foods)	6 mo	*n* = 266 men with PSA concentrations of 2.0–2.95 ng/mL or 3.0–19.95 ng/mL and negative prostate biopsies	1. Green tea drink (3 cups, unblinded) (*n* = 45) 2. Green tea caps (blinded, 600 mg flavan-3-ol EGCG) (*n* = 45) 3. Placebo (*n* = 43) 4. Lycopene-rich foods (unblinded) (*n* = 44) 5. Lycopene (15 mg/d) caps (blinded) (*n* = 44) 6. Placebo (*n* = 45)	PSA concentrations did not differ between lycopene, green tea, or placebo groups at 6 months.
Morgia ^Š^ [164]	IT	Double-blind	1 yr	*n* = 225 patients who underwent prostate biopsy when PSA ≥ 4 ng/mL, and/or suspicion of PCa	1. Saw palmetto (320 mg), Se and lycopene (*n* = 75) 2. Tamsulosin (0.4 mg) (*n* = 75) 3. Saw palmetto (320 mg), Se, lycopene, and tamsulosin (0.4 mg) (*n* = 75)	No differences in terms of mean changes in PSA between the groups were noted.
Suardi [169]	IT	Double-blind	3 mo before surgery	*n* = 36 patients with BPH and obstructive symptoms, scheduled for surgery	1. Saw palmetto, quercetin and β-sitosterol (*n* = 18) 2. No intervention (*n* = 18)	No differences were noted in the PSA of the two groups.
Morgia [168]	IT	NR	6 mo	*n* = 168 men with histological PCI diagnosis associated with BPH, HGPIN, and/or ASAP, and suspected PCa	1a. Saw palmetto, Se and lycopene (*n* = 54) 1b. No intervention (*n* = 54) 2a. Saw palmetto, Se, and lycopene + a-blockers (*n* = 30) 2b. No ONS or a-blockers (*n* = 30)	Mean PSA was reduced in group 1a as compared with the controls (1b).
Ide [170]	JP	Double-blind	6 mo	*n* = 100 men undergoing systematic prostate biopsy for elevated PSA, without PCa or PIN diagnosis	1. Curcumin (100 mg/d) + isoflavones (40 mg) (*n* = 50) 2. Placebo (*n* = 50)	PSA concentrations decreased in patients with baseline PSA ≥ 10 treated with isoflavones and curcumin.
Meyer [175]	CA	Double-blind	8 yrs	*n* = 5034 men (45–60 yrs)	1. Vitamin C (120 mg), α-tocopherol (30 mg), β-carotene (6 mg), Se (100 μg), and Zn (20 mg) (*n* = 2522) 2. placebo (*n* = 2512)	Supplementation did not affect PSA concentrations. Among men with normal PSA on ONS, a reduction in the rate of PCa was noted. In those with elevated PSA at baseline, ONS was associated with an increased PCa incidence of borderline significance.
Klein [61]	US	Double-blind	7–12 yrs	*n* = 34,887 men with PSA ≤ 4.0 ng/mL, a DRE not suspicious for PCa, and age ≥ 50 yrs (black men) and ≥ 55 yrs (all others)	1. Se (200 μg/d) (*n* = 8752) 2. Vitamin E (400 IU/d) (*n* = 8737) 3. Se (200 μg) + Vitamin E (400 IU) (*n* = 8702) 4. Placebo (*n* = 8696)	2/3 of the men in each of the 4 groups had elevated PSA (NS). No difference was observed in the PSA velocity each consecutive year between groups. Vitamin E ONS increased the risk of PCa.
Marks [126]	US	Double-blind	6 mo	*n* = 44 men (45–80 yrs) with symptomatic BPH	1. Saw palmetto (106 mg) + herbs (nettle root, pumpkin) (*n* = 21) 2. Placebo (*n* = 23)	A lack of a change in serum PSA was noted.
Preuss [171]	US	Double-blind	3 mo	*n* = 127 men with BPH	1. Cernitin, saw palmetto, B-sitosterol, vitamin E (*n* = 70) 2. Placebo (*n* = 57)	The PSA scores showed no differences when comparing the intervention and placebo groups.

AS, active surveillance; ASAP, atypical small acinar proliferation; BPH, benign prostate hyperplasia; CI, confidence intervals; CoQ10, coenzyme Q10; DES, Diethylstilbestrol; DRE, digital rectal examination; EGCG, (-)-epigallocatechin-3-gallate; GTCs, green-tea catechins; HGPIN, high grade prostatic intraepithelial neoplasia; HR, hazard ratio; IU, international units; LUTS, lower urinary tract symptoms; MC, multi-county; mHGPIN, multifocal high grade prostatic intraepithelial neoplasia; NS, not significant; PCa, prostate cancer; PCI, prostatic chronic inflammation; PIN, prostatic intraepithelial neoplasia; *PSA*, prostate-specific antigen; PSADT, prostate-specific antigen doubling time; RCT, randomized controlled trials; Se, selenium; Zn, zinc. ^†^ phase II trial; ^Š^ post-hoc analysis; ^‡^ cross-over trial.

**Table 10:** RCTs examining the effect of fatty acids supplementation, or frequent intake of foods rich in fatty acids, on serum PSA concentrations among men with PCa or increased PCa risk.

First Author	Origin	Masking	Duration	Patients	Interventions	Results
Chan [196]	US	Double-blind	3 mo	*n* = 84 men with low-burden PCa, choosing AS for disease management	1. 3 × 1 g fish oil caps/d (1098 mg EPA + 549 mg DHA) (*n* = 27) 2. 2 × 15 mg lycopene caps/d (*n* = 29) 3. Placebo (*n* = 28)	No difference was observed in Δ PSA concentrations post-intervention, between lycopene or fish oil.
Higashihara [197]	JP	NR	2 yrs	*n* = 62 men with PCa and post-surgical PSA < 0.2 ng/mL at 3 mo	1. EPA (2.4 g/d) (*n* = 32) 2. Control group without intervention (*n* = 30)	The recurrence-free survival rate did not differ between groups.
Simon ^‡^ [199]	US	NR	6 mo each arm (4 mo washout)	*n* = 40 middle-aged men	1. Walnut consumption (35 g/d, 12% TEI) (*n* = 40) 2. No ONS (*n* = 40)	No difference was observed in the PSA concentrations.
Spaccarotella ^‡^ [198]	US	NR	8 wks	*n* = 21 men at risk for PCa	1. Usual diet + walnut ONS (75 g/d) isocaloric to habitual diet (*n* = 21) 2. Usual diet, no ONS (*n* = 21)	A linear mixed model revealed that, although PSA was unchanged, the ratio of free:total PSA was increased.
Brouwer [200]	NL	Double-blind	40 mo	*n* = 1622 men with a history of a MI (60–80 yrs) with an initial PSA < 4 ng/mL	1. ALA (2 g/d) in margarine spreads (*n* = 807) 2. Placebo in margarine spreads (*n* = 815)	Mean serum PSA increased by 0.42 ng/mL in the placebo group and by 0.52 ng/mL in the ALA group (NS).
Hamazaki [201]	JP	NR	12 wks	*n* = 20 men (>50 years old) without PCa diagnosis	1. EPA (2.4 g/d) (*n* = 10) 2. No intervention (*n* = 10)	No differences were observed in the PSA concentrations between the two groups.

ALA, alpha-linolenic acid; AS, active surveillance; CI, confidence intervals; CHO, carbohydrates; CoQ10, coenzyme Q10; DHA, docosahexaenoic acid; EPA, eicosapentaenoic acid; GLA, γ-linolenic acid; LFFO, low fat/fish oil; MI, myocardial infarction; NR, not reported; NS, not significant; PCa, prostate cancer; PRO, proteins; PSA, prostate-specific antigen; RCT, randomized controlled trials; TEI, total energy intake. ^‡^ cross-over trial.

**Table 11:** RCTs examining the effect of dietary patterns on serum PSA concentrations among men with PCa or increased PCa risk.

First Author	Origin	Masking	Duration	Patients	Interventions	Results
Hébert [206]	US	Open-label	6 mo	*n* = 54 men with a confirmed PCa, treated by prostatectomy, or radiation therapy	1. Diet (low meat and dairy, increased intake of whole grains, soybeans and by-products, other beans, and vegetables), PA (45-min sessions), and stress reduction sessions (*n* = 29) 2. Usual treatment (*n* = 25)	No difference in Δ PSA was noted by intervention status. Men increasing their fruit intake experienced no PSA rise.
Demark-Wahnefried [208]	US	Single-blind	until prostatectomy (30.7 d)	*n* = 161 scheduled at least 21-d before prostatectomy	1. Usual diet (*n* = 41) 2. Flaxseed-supplemented diet (30 g/d) (*n* = 40) 3. LFD (<20% fat) (*n* = 40) 4. Flaxseed-supplemented (30 g/d) LFD (<20% fat) (*n* = 40)	Over the presurgical study period serum PSA decreased in all arms, with no differences in change observed between arms.
Ornish [210]	US	NR	1 yr	*n* = 98 men with early, biopsy-proven PCa after 1 yr, PSA 4–10 ng/mL and Gleason score ≤ 7	1. Intensive lifestyle program promoting a vegan diet, supplemented with soy (1 serv of tofu + 58 g fortified soy PRO drink), fish oil (3 g/d), vitamin E (400 IU/d), Se (200 μg/d), and vitamin C (2 g/d), moderate aerobic PE (walking 30 min, 6 d/wk), stress management (yoga-based stretching, breathing, meditation, imagery, relaxation for a 60 min/d), and 1-h support group once weekly to enhance adherence to the intervention. Diet: fruits, vegetables, whole grains (complex CHO), legumes, and soy products, low in simple CHO and with 10% fat (*n* = 44) 2. Usual diet (*n* = 49)	Changes in serum PSA from baseline to 12 mo were different between groups, with favorable changes in the experimental group. Serum PSA decreased (0.25 ng/mL, or 4%) from baseline in the treatment arm, but increased in the control group.
Kellogg Parsons [211]	US	Single-blind	2 yr	*n* = 443 men (50–80 yrs) with biopsy-proven PCa	1. Counseling behavioral intervention by phone promoting the intake of ≥7 vegetable serv/d (*n* = 226) 2. Written information about diet and PCa (*n* = 217)	There were no significant differences in TTP between groups.
Aronson ^†^ [212]	US	Single-blind	4–6 wks	*n* = 55 patients undergoing radical prostatectomy	1. Western diet (40% fat, 15% PRO, 45% CHO, 15 g fiber/d, n-6:n-3 FA ratio of 15:1) (*n* = 26) 2. LFFO diet (15% fat, 15% PRO, 70% CHO), 39 g fiber/d and 5 × 1.1 g fish oil caps/d (200 mg EPA, 367 mg DHA) with a ratio of n-6:n-3 FA to 2:1 (*n* = 26)	No differences were noted in the PSA concentrations of participants in the two groups.
Aronson [213]	US	NR	4 wks	*n* = 18 men with PCa who did not receive prior therapy	1. High-fiber LFD (15% fat, 30% PRO, 55% CHO), 35 g soy PRO/d, and 35 g fiber/d) (*n* = 9) 2. Western diet (40% fat, 30% PRO, no soy, 30% CHO, 10 g fiber/d) (*n* = 9)	No differences were observed in the PSA concentrations.
Antwi [142]	US	NR	6 mo	*n* = 54 men with a history of PCa and rising PSA concentrations post-prostatectomy/radiation	1. Dietary modifications, PA, and mindfulness-based stress reduction training, including shopping guidelines (*n* = 29) 2. Standard care (*n* = 25)	No differences were observed in the PSA concentrations between participating groups.
Tariq [223] ^‡^	CA	NR	4 mo	*n* = 14 healthy men with hyperlipidemia	1. Diet high in soluble fiber (approx. 25–30 g fiber/1000 kcal, ≤20% fat, ≤20% PRO, ≥60% CHO) (*n* = 9) 2. Diet high in insoluble fiber (approx. 25–30 g fiber/1000 kcal, ≤20% fat, ≤20% PRO, ≥60% CHO) (*n* = 5)	Serum PSA concentration was lower with the soluble than the insoluble fiber diet.
Freedland [217]	US	NR	6 mo	*n* = 42 patients with PCa initiating ADT	1. LCD (≤20g CHO/d) plus walking (≥30 min for ≥5 d/wk) 2. Usual diet and exercise patterns	No differences were observed in the PSA concentrations.
Freedland [218]	US	NR	6 mo	n = 34 men with PCa and BCR after local treatment	1. LCD (≤20g CHO/d) (*n* = 14) 2. Usual diet (*n* = 20)	PSA values did not differ between groups. The proportion of patients with slowed PSADT was greater in the LCD arm.
Li [219]	US	NR	4 yr	*n* = 40 men post-prostatectomy, at high risk for recurrence	1. LFD (15% fat), high-fiber (18 g/1000 kcal) diet supplemented with 40 g soy PRO + individual counseling sessions (*n* = 26) 2. USDA recommended diet (*n* = 14)	No significant changes in PSA were reported between groups.
Carmody [220]	US	NR	3 mo	*n* = 24 men previously treated for PCa and their partners	1. 11 dietary and cooking classes (emphasizing plant-based foods and fish -salmon-, vegetables-, cruciferous varieties-, and whole grains, as well as soy foods, with avoidance of meat, poultry, and dairy) (*n* = 10) 2. Control group (*n* = 14)	No change was found in the rate of PSA increase between the two groups; the mean PSADT for the intervention participants was substantially longer.
Shike [221]	US	NR	2 yrs	*n* = 1230 men with normal DRE results, PSA concentrations < 3 ng/mL	1. Intensive counseling towards an LFD, high in fiber, fruits, and vegetables (*n* = 627) 2. Standard brochure on a healthy diet (*n* = 603)	No difference was observed in the distributions of the PSA slopes, the PSA slopes per se, or the % of high PSA concentrations between groups. The incidence of PCa at 4 yrs was similar.
Eastham ^Š^ [222]	US	Open-label	4 yrs	*n* = 1197 men	1. LFD high in fiber, fruits, and vegetables (*n* = 611) 2. Usual diet (*n* = 586)	No difference was noted in serum PSA concentrations by dietary intervention.

ADT, androgen deprivation therapy; ALA, alpha-linolenic acid; AS, active surveillance; BCR, biochemical recurrence; CI, confidence intervals; CHO, carbohydrates; CoQ10, coenzyme Q10; DHA, docosahexaenoic acid; DRE, digital rectal examination; EPA, eicosapentaenoic acid; FA, Fatty acids; GLA, γ-linolenic acid; LCD, Low-carbohydrate diet; LFD, Low-fat diet; LFFO, low fat/fish oil; NR, not reported; NS, not significant; PCa, prostate cancer; PE, physical exercise; PRO, proteins; PSA, prostate-specific antigen; PSADT, prostate-specific antigen doubling time; RCT, randomized controlled trials; TEI, total energy intake; TTP, time to progression (PSA ≥ 10 ng/mL); USDA, United States Department of Agriculture. ^†^ phase II trial; ^‡^ cross-over trial; ^Š^ post-hoc analysis.
